# Comparative Outcomes of Drug-Eluting Stents Versus Drug-Eluting Balloons in Patients with Chronic Kidney Disease: A Systematic Review of Current Evidence

**DOI:** 10.12688/f1000research.177235.1

**Published:** 2026-02-18

**Authors:** Isnaini Isnaini, Bayu Satria Wiratama, Muhammad Syairaji, Soraya Isfandiary Iskandar, alya Nur Fadhilah, I Gde Rurus Suryawan, Yudi Her Oktaviono

**Affiliations:** 1 Department of Cardiology and Vascular medicine, Universitas Airlangga Departemen Kardiologi dan Kedokteran Vaskular, Surabaya, East Java, Indonesia; 2 Department of Biostatistics Epidemiology and Population Health, Gadjah Mada University Department of Biostatistics Epidemiology and Population Health, Yogyakarta, Special Region of Yogyakarta, Indonesia; 3 Department of Health Information and Services, Universitas Gadjah Mada Vocational College, Yogyakarta City, Special Region of Yogyakarta, Indonesia

**Keywords:** Chronic Kidney Disease; Percutaneous Coronary Intervention; Drug-Eluting Stents; Drug-Eluting Balloons; Coronary Artery Disease; Cohort Study

## Abstract

**Background:**

Chronic kidney disease (CKD) is a significant comorbidity in cardiovascular treatments, increasing the likelihood of adverse outcomes. Choosing between drug-eluting balloons (DEB) and drug-eluting stents (DES) in patients with CKD requires careful consideration of efficacy and safety. To address this gap, we conducted a systematic review of DEB and DES in CKD patients, focusing on mortality, MACE, TLR, and stent thrombosis.

**Methods:**

This systematic review followed the PRISMA guidelines. Comprehensive searches were performed in PubMed, Cochrane Library, Scopus, Web of Science, and ScienceDirect until September 2025. Randomized controlled trials (RCTs) and cohort study comparing DEB and DES in CKD patients were included. Four reviewers independently screened and extracted data on mortality, MACE, target lesion revascularization (TLR), and stent thrombosis, and conflicts were resolved through discussion.

**Results:**

Five studies (four retrospective cohorts, one RCT) met inclusion criteria after screening 4,093 records, involving CKD/ESRD patients undergoing PCI with DEB (paclitaxel-coated) versus contemporary DES. MACE rates were equivalent: 17% in both arms (Funayama et al., ESRD dialysis cohort) and 10% in both arms (Jeger et al., RCT; HR 0.99, 95% CI 0.38-2.57). Target lesion revascularization (TLR) and target vessel failure (TVF) showed heterogeneity: DES favored in some (e.g., Waha et al., aHR 0.20, 95% CI 0.06-0.69), DEB numerically in others (e.g., Joh et al., 2-year TVF 14.3% DEB vs. 27.8% DES), with no meta-analysis due to clinical/methodological differences; thrombosis was low and sparse (0% DEB vs. 2.2% DES in Funayama).

**Conclusion:**

In CKD/ESRD patients, DEB and DES yield equivalent MACE, though TLR/TVF results vary by study design, CKD severity, and procedural factors. No clear superiority emerged, supporting individualized strategy selection based on lesion characteristics, bleeding risk, and expertise. Prospective RCTs with standardized CKD subgroups and DEB optimization are needed for definitive guidance.

PROSPERO registration ID: CRD420251039001 (05/15/2025)

## Introduction

The patients with chronic kidney disease were older and had a greater number of risk factors linked to cardiac events and mortality, including hypertension, heart failure, and diabetes mellitus. In this study, advanced age and traditional risk factors significantly influenced the adverse cardiovascular outcomes observed in individuals with CKD. ^1^ Renal failure is a recognized risk factor for ischemic heart disease, linked to accelerated atherosclerosis, endothelial dysfunction, oxidative stress, and inflammation. These factors lead to the development of coronary artery atherosclerosis and elevated cardiovascular mortality. ^2, 3^ Moreover, individuals with chronic kidney disease (CKD) who underwent percutaneous coronary intervention (PCI) with drug-eluting stents (DES) exhibited inferior post-procedural cardiovascular outcomes compared to those without CKD, characterized by increased mortality, hemorrhagic complications, and extended hospital stays. ^3, 4^

Drug-eluting stents (DES) remain the standard of treatment for patients undergoing percutaneous coronary intervention (PCI). ^5, 6^ However, DES are associated with a gradually increasing and permanent risk of adverse events, particularly due to late stent thrombosis and in-stent restenosis, which has an incidence rate of 2% per year without any observed plateau. ^6^ This risk is even higher when complex and long lesions are treated. ^7^ Drug-eluting balloons (DEB) have recently come to light as a possible alternative to DES. Following adequate lesion preparation, unlike traditional stents, DEBs can release an antiproliferative drug into the vessel wall without leaving behind a permanent metal scaffold.

Data from the Korean Multicenter In-Stent Restenosis Registry indicate that whereas rates of target lesion failure were reduced in modern drug-eluting stents (DES) compared to drug-eluting balloons (DEB), this disparity diminished in patients with chronic kidney disease (CKD), defined as an estimated glomerular filtration rate (eGFR) < 60 ml/min/1.73 m ^2^. ^8^ The previous systematic review showed that drug-coated balloons exhibited comparable risks of major adverse cardiovascular events (MACE) to drug-eluting stents in the context of ST-elevation myocardial infarction (STEMI). ^9^ In patients with de novo large vessel coronary artery disease, the utilization of DEB compared to DES is linked to a similar risk of TLR; nevertheless, DES produces superior late angiographic outcomes. ^10^ With the advent of drug-eluting balloons (DEB) as an alternative, it is essential to compare the outcomes between DES and DEB in CKD patients for all durations of follow-up to provide clearer clinical guidance. Therefore, the purpose of this study was to compare the outcomes of these two approaches in CKD patients by conducting a systematic review.

Comparative studies of DEB and DES in patients with chronic kidney disease (CKD) found that clinical outcomes were generally equal across multiple outcomes. Multiple studies have found that DEB and DES had similar rates of major adverse cardiovascular events (MACE), especially in individuals with small vessel or de novo coronary artery disease. ^11– 13^ Similarly, target lesion revascularization (TLR) rates were largely comparable among studies, with DEB showing reduced TLR rates in small vessel disease. ^14, 15^ Furthermore, late lumen loss was much lower with DEB than with DES, ^12, 13, 15^ indicating possible advantages in maintaining long-term vessel patency. Importantly, DEB-treated patients had fewer major bleeding events, ^11^ which is critical considering the increased bleeding risk associated with uremic platelet dysfunction in CKD. Mortality rates, both all-cause and cardiovascular, were shown to be equal across the two methods. ^11– 13^

Clinically, these data suggest that DEB is a viable and safe alternative to DES in some CKD populations, notably those with small artery or de novo lesions and patients at high risk of bleeding. However, long-term data are still scarce, and the long-term viability of DEB results beyond the medium-term follow-up period is unknown. Furthermore, despite revascularization, hemodialysis-dependent CKD patients continue to have poor outcomes, indicating the need for customized treatment regimens and improved medical care. Despite growing data, the long-term efficacy and safety of DEB over DES in CKD populations, especially across renal dysfunction stages, are still questionable. The objective of this study was to compare the outcomes of these two procedures in individuals with chronic kidney disease through a systematic review.

## Methods

This systematic review was conducted according to the Preferred Reporting Items for Systematic Reviews and Meta-Analyses (PRISMA) 2020 statement and registered pro-spectively with PROSPERO (CRD420251039001). The protocol was developed a priori to minimize bias and ensure transparency in the review process, following the Cochrane Handbook for Systematic Reviews of Interventions.

### Eligibility criteria

**Study designs**

We included Randomized Controlled Trials (RCTs) and non-randomized compara-tive studies (prospective or retrospective cohort studies) that directly compared drug-eluting balloons (DEB) with drug-eluting stents (DES) in the target population. RCTs were prioritized for inclusion due to their superior methodological rigor in minimizing selection and performance bias. Non-randomized studies were included to maximize clinical relevance and capture real-world evidence, particularly for populations or sce-narios underrepresented in RCT data. We excluded case reports, case series, single-arm studies, editorials, commentaries, and conference abstracts without subsequent peer-reviewed publication to ensure methodological quality and minimize reporting bias.

### Participants

We included studies enrolling adults (≥18 years) with chronic kidney disease (CKD) undergoing percutaneous coronary intervention (PCI). CKD was defined according to the classification used in individual studies, with particular focus on the most common definition of estimated glomerular filtration rate (eGFR) <60 mL/min/1.73 m ^2^ and/or dialysis dependence. Studies with mixed or heterogeneous populations were included only if separate outcome data for the CKD subgroup were reported or if ≥80% of participants met CKD criteria. We excluded studies that did not provide specific CKD data or where CKD patients represented <20% of the study population, as these would not adequately address our research questions specific to this vulnerable population.

### Interventions and comparators

The intervention of interest was any drug-eluting balloon (DEB), regardless of drug agent (e.g., paclitaxel, sirolimus), coating technology, or balloon brand. The comparator was drug-eluting stents (DES) of any generation (first-generation sirolimus-eluting, paclitaxel-eluting; second-generation everolimus-eluting, zotarolimus-eluting) or newer generations. We excluded studies comparing DEB with bare-metal stents (BMS) alone, as BMS is no longer standard of care and would not reflect contemporary clinical practice.

### Outcomes

Primary outcomes included: (1) all-cause mortality (combining cardiovascular and non-cardiovascular deaths); (2) major adverse cardiovascular events (MACE), defined as the composite of death (all-cause or cardiovascular), myocardial infarction (MI), and target lesion/vessel revascularization (TLR/TVR).

Secondary outcomes included: (1) cardiovascular-specific mortality; (2) target lesion revascularization (TLR), defined as any repeat revascularization procedure of the index lesion due to symptoms, objective evidence of ischemia, or restenosis ≥50%; (3) target vessel failure (TVF), defined as cardiac death, MI, or TVR in the target vessel; (4) stent thrombosis (for DES arm); (5) adverse events including clinically significant bleeding, stroke, and infection.

Data were collected for the longest available follow-up period reported in each study, as well as at standardized intervals (6, 12, 24, and 36 months) when available to enable subgroup temporal analyses.

### Exclusion criteria

We excluded studies meeting any of the following criteria: (1) non-English language publications (due to resource constraints); (2) no specific comparison between DEB and DES; (3) absence of outcome data relevant to our primary or secondary outcomes; (4) studies with <5 participants per arm, as these lack adequate statistical power; (5) studies with no CKD-specific data or in populations without CKD; (6) publications that were review articles, conference proceedings without full-text availability, or gray literature without peer-review. This approach ensures methodological quality while being pragmatic about available resources.

### Information sources

**Database searching**

We conducted a comprehensive systematic search of five major electronic databases from inception through September 2025: PubMed/MEDLINE, Scopus, Web of Science, and ScienceDirect. These databases were selected to capture the breadth of published literature across medical and biomedical databases, ensuring comprehensive coverage of indexed publications. No language restrictions were applied during the initial search to maximize sensitivity, though we subsequently limited inclusion to English-language publications due to resource constraints. The initial search yielded 4,093 records (PubMed n=530, Scopus n=2,245, Web of Science n=909, ScienceDirect n=409), demonstrating the substantial literature landscape on this topic.

To minimize publication bias and capture completed but unpublished studies, we systematically searched grey literature and other sources:

• Reference lists of all included studies and related systematic reviews were hand-screened by two independent reviewers to identify potentially missed citations
• Citation tracking using forward citation searching in Google Scholar for studies citing included articles
• Author contact: For studies with incomplete data reporting, we contacted corresponding authors to request unpublished outcome data

This comprehensive grey literature approach helps reduce potential bias toward published studies with statistically significant findings.

### Search strategy

**Search string development**

We developed a comprehensive search strategy in collaboration with a medical librarian using controlled vocabulary (Medical Subject Headings—MeSH terms) combined with free-text terms to capture variations in terminology and nomenclature. The search strategy incorporated three conceptual domains:

a. Domain 1 - CKD Population: (“Chronic Kidney Disease” OR CKD OR “renal insufficiency” OR “renal impairment” OR “kidney failure” OR “renal failure”)
b. Domain 2 - Interventions: ((“Drug-Eluting Balloon” OR “Drug-Coated Balloon” OR DEB OR DCB OR “drug coated balloon” OR “drug eluting balloon”) OR (“Drug-Eluting Stent” OR DES OR “drug eluting stent”))
c. Domain 3 - Clinical Context & Outcomes: (MACE OR TLR OR TVR OR “target vessel” OR “target lesion” OR “major adverse cardiac event” OR mortality OR revascularization OR “myocardial infarction”).

Truncation and boolean operators were applied systematically to ensure sensitivity. The complete search strategies for each database, including MeSH terms specific to each platform, are detailed in Supplementary Material 1. Notably, search strategies were adapted to accommodate database-specific syntaxes and indexing practices (e.g., PubMed uses MeSH subject headings, while Scopus uses Emtree descriptors).

**Search strategy validation**

To ensure search strategy sensitivity and specificity, we performed preliminary searches and validated the strategy by confirming that known relevant studies (identified through preliminary scoping) were retrieved. This validation process helps ensure the search was comprehensive without excessive retrieval of irrelevant citations.

### Selection process

**Screening and deduplication phase**

All retrieved records were imported into rayyan.ai (a web-based systematic review management platform) for automated and manual deduplication. Title and Abstract Screening: Four independent reviewers (BSW, MS, SII, ANF), trained using a stand-ardized screening manual, independently reviewed titles and abstracts of all articles using rayyan.ai. A broad inclusion approach was used at this stage to maximize sensitivity and minimize the risk of excluding relevant studies. Any article deemed potentially relevant by any reviewer proceeded to full-text review. Full text review conducted ac-cording to prespecified inclusion and exclusion criteria. Authors documented reasons for exclusion such as wrong population, wrong intervention/comparison, wrong outcome measure, no comparative data, wrong publication type, unclear intervention, and full-text unavailable despite contact attempts. This transparent documentation of exclusion reasons facilitates assessment of review bias and allows future researchers to identify studies excluded for potentially remediable reasons. All studies which met all eligibility criteria were included in the qualitative synthesis.

**Agreement and conflict resolution**

Disagreements between reviewers at the full-text stage were resolved through discussion and consensus. When consensus could not be reached, a senior adjudicator (the primary investigator or senior methodologist) made the final decision. This hierarchical resolution process balances reviewer autonomy with the need for consistent application of eligibility criteria. All disagreements and their resolutions were documented in a standardized form to provide transparency regarding how methodological decisions were made and to identify patterns in reviewer agreement for future investigator training.

### Data collection process

**Data extraction**

Data extraction was performed independently and in duplicate by four trained reviewers using a pre-piloted, standardized data extraction form programmed into Rayyan.ai. Before extracting data from included studies, reviewers pilot-tested the form on 2-3 representative studies to ensure consistency of interpretation and completeness of the form.

Type of Data Extracted

Study-Level Characteristics:

• Author name, publication year, journal
• Country/countries of study conduct
• Study design classification (RCT, prospective cohort, retrospective cohort)

Participant Characteristics:

• Total sample size (and N per study arm)
• Age (mean, median, range)
• Sex distribution (% male)
• CKD stage/severity definition and distribution (eGFR <60, on dialysis)

Intervention Characteristics:

• DEB type, brand, and drug agent (paclitaxel, sirolimus, other)
• Drug dose and coating technology
• DES type, generation, and drug agent

Outcome Data:

• Effect estimates (Hazard Ratios, Risk Ratios, Odds Ratios with 95% confidence intervals)
• P-values or test statistics

**Missing data management**

When outcome data were incomplete or unclear, reviewers contacted corresponding authors via email with specific requests for unpublished or supplementary data. Requests included patient-level data when available, event counts by follow-up period, and clarification of outcome definitions. We allowed 4 weeks for author responses before proceeding with available data. The frequency and nature of missing data from author contact attempts were documented.

**Data extraction agreement**

Discrepancies between independent extractions were identified through comparison and resolved through discussion and consensus. Any unresolved discrepancies were adjudicated by a senior reviewer. The percentage agreement and specific categories with high disagreement rates were documented to inform quality assurance.

### Data items

Extracted data were organized into two main evidence tables:

Table 1 about study Characteristics consist of summarizes study design, setting, participant demographics, CKD criteria, and comorbidity profiles. This table enables readers to assess population applicability and identify sources of heterogeneity.

**Table 1.** Study characteristics.

Study ID	Author(s) & Year	Country	Study design	Sample size (N)	Mean age (years)	Male (%)	CKD/ESRD definition	CKD/ESRD proportion	Risk of bias level
1	Funayama et al., 2023	Japan	Retrospective cohort	400	69.1 ± 10.1	76.3	ESRD-Dialysis	ESRD 100%	Moderate
2	Lee et al., 2024	South Korea	Retrospective cohort	628	65.6 ± 10.0	68.2	eGFR <60 ml/min ^1^	CKD 28.3%	Moderate
3	Wańha et al., 2024	Poland	Retrospective cohort	846	Not specified	Not specified	Not specified	CKD 18.9%	Moderate
4	Jeger et al., 2018	Switzerland	RCT (non-inferiority)	758	67.2 ± 10.3	73.5	Not specified	Renal failure 14.9%	Low
5	Joh et al., 2024	South Korea	Retrospective cohort	1,616	64.2 ± 11.2	78.4	Not specified	CKD 6.7%	Moderate

^1^ per 1.73 m ^2^ calculated using 4-component Modification of Diet in Renal Disease study (MDRD) equation incorporating age, race, sex, and serum creatinine.

Table 2 about intervention and Outcome Data: Presents specific details of the DEB and DES interventions (type, drug, dosing), clinical outcomes with event counts and effect estimates (HR/RR/OR with 95% CI), and follow-up durations. This facilitates outcome synthesis and identification of heterogeneity sources.

**Table 2.** Study intervention characteristics.

Study ID	Author(s) & Year	DES group (n)	DEB group (n)	DES type	DEB type	Outcome
1	Funayama et al., 2023	312	88	New generation DES	paclitaxel-coated balloon	TLR: DES 14.1% vs DEB 14.7%, P=0.864 Target lesion thrombosis: 0% DEB vs 2.2% DES (7 patients) MACE: 17% DES vs 17% DEB; no significant DES–DEB difference
2	Lee et al., 2024	409	219	Everolimus eluting stent or zotarolimus eluting stent	paclitaxel-coated balloons	TLF: 13.7% DES vs 26.9% DEB; aHR 0.60 (95% CI 0.27-1.33)
3	Wańha et al., 2024	381	465	thin-strut (<100 μm) new-generation DES	paclitaxel-coated balloons	TLR: DES vs DEB; aHR 0.2 (0.06-0.69)
4	Jeger et al., 2018	376	382	second-generation Xience (everolimus-eluting) or Taxus Element (paclitaxel-eluting)	paclitaxel-coated balloon	MACE: 10% DES vs 10% DEB; HR 0.99 (95% CI 0.38-2.57)
5	Joh et al., 2024	1478	138	New generation DES	paclitaxel-coated balloon	2 year TVF: 27.8% DES vs 14.3% DEB; HR 2.5 (95% CI 0.29-20)

### Study risk of bias assessment

**Assessment tools and approach**

All included studies (n=5) underwent independent duplicate risk-of-bias (RoB) assessments by four trained reviewers using validated Cochrane tools. For the RCT (n=1), Cochrane RoB 2 evaluated five domains—randomization process, deviations from intended interventions, missing data, outcome measurement, and selective reporting—judged as low risk, some concerns, or high risk; overall risk categorized accordingly (low if all low; some concerns/high otherwise). For non-randomized studies (n=4), ROBINS-I assessed seven domains—confounding, selection, intervention classification, deviations from intended interventions, missing data, outcome measurement, and selective reporting—rated low/moderate/serious/critical risk, with overall risk determined algorithmically from domain judgments.

**Disagreement resolution**

Disagreements in bias assessment between reviewers were resolved through discussion and consensus. Persistent disagreements were adjudicated by a senior investigator (primary investigator or experienced methodologist).

### Effect measures

Not applicable. Due to substantial heterogeneity in study design, CKD definitions, intervention types, and outcome measurement timing (see Section 2.9), quantitative meta-analysis was deemed inappropriate. Therefore, no effect measures were pre-specified for meta-analysis. However, we systematically extracted and presented effect estimates (Hazard Ratios, Risk Ratios, Odds Ratios with 95% confidence intervals) from individual studies in evidence tables to facilitate narrative synthesis.

### Synthesis methods

We conducted a narrative synthesis instead of quantitative meta-analysis due to substantial clinical, methodological, statistical heterogeneity, and limited included studies (n=5), which precluded valid pooled estimates, subgroup analyses, or meta-regression. Clinical heterogeneity arose from variations in CKD definitions (e.g., eGFR cutoffs vs. dialysis status), severity/comorbidity profiles, target lesion characteristics (vessel size, complexity, location), and interventions (DES generations, DEB agents). Methodological differences included mixed designs (RCTs vs. observational cohorts), inconsistent confounder adjustment, and unharmonized risks of selection bias/confounding, while statistical heterogeneity involved divergent outcome definitions/timing, follow-up durations (6 months to >3 years), and composite outcome reporting.

The narrative synthesis employed a structured, pre-specified, reproducible approach per the registered protocol, integrating evidence tables, descriptive comparisons, bias assessments, consistency evaluations, and meta-narrative synthesis to address heterogeneity constraints. Evidence tables ( Tables 1- 2) systematically organized study characteristics (designs, populations, interventions) and outcomes for visual pattern/anomaly identification. Descriptive comparisons qualitatively assessed outcome findings, effect directions/magnitudes with 95% CIs, while integrating RoB assessments via sensitivity analyses (low- vs. high-bias studies) and discussions of bias influences. Consistency evaluations examined directional agreement, effect magnitude overlap in CIs, and outliers; meta-narrative elements explained heterogeneity sources, clinical implications, and contextualized findings against existing literature/guidelines.

## Results

### Study selection

**Figure 1.**
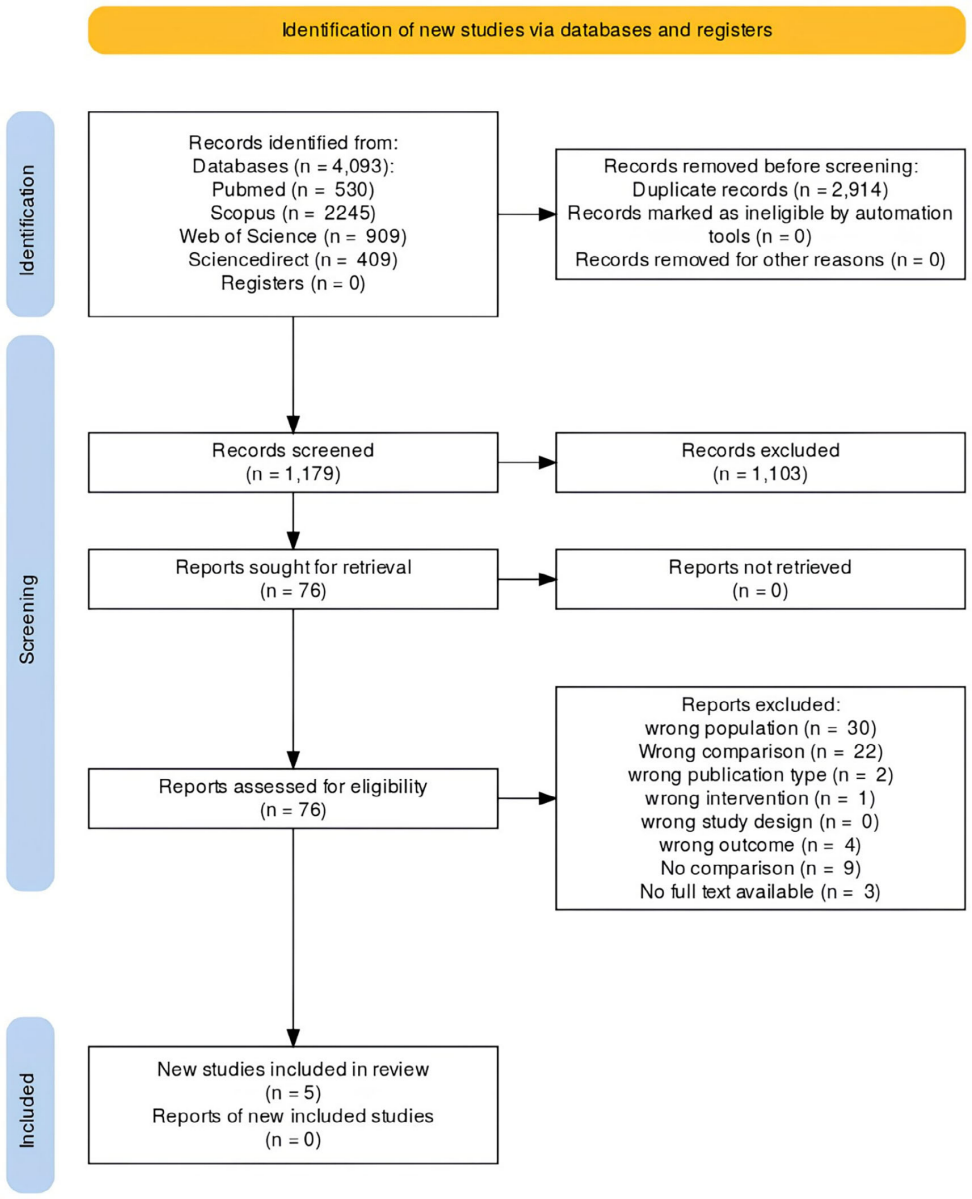
PRISMA flowchart.

A total of 4,093 records were identified through database searching (PubMed 530, Scopus 2,245, Web of Science 909, ScienceDirect 409) as showed in Figure 1. After removal of 2,914 duplicates, 1,179 unique records underwent title and abstract screening. Of these, 1,103 records were excluded as clearly not meeting the eligibility criteria. Seventy six full text articles were retrieved and assessed for eligibility, resulting in exclusion of 71 reports for the following reasons: wrong population (n = 30), wrong comparison (n = 22), wrong publication type (n = 2), wrong intervention (n = 1), wrong outcome (n = 4), no comparison group (n = 9), and no full text available (n = 3). Five studies met all inclusion criteria and were included in the final qualitative synthesis.

### Study and patient characteristics

Five comparative studies (four retrospective cohort studies and one randomized controlled trial) comprising CKD or ESRD populations undergoing PCI with DEB versus DES were included ( Table 1). The studies were conducted in Japan, South Korea, Poland (multicenter), and Switzerland (multicenter), reflecting predominantly East Asian and European healthcare settings. Total sample sizes ranged from 400 to 1,616 participants, with mean ages between 64.2 and 69.1 years where reported, and a consistently high proportion of male patients (68.2%–78.4%).

One study exclusively enrolled ESRD patients on dialysis (Funayama et al., 2023), whereas others included broader CKD populations defined by reduced eGFR or reported as “renal failure.” In Lee et al. (2024), CKD was explicitly defined as eGFR < 60 ml/min per 1.73 m ^2^ based on the four component MDRD equation, with CKD present in 28.3% of the cohort. The proportion of patients with CKD or ESRD ranged from 6.7% in the largest South Korean cohort (Joh et al., 2024) to 100% in the dialysis only Japanese cohort, with intermediate values of 14.9% and 18.9% reported in the Swiss and Polish multicenter studies, respectively.

### Intervention characteristics

Across all five studies ( Table 2), the comparator arm used contemporary new generation or second generation DES, while the intervention arm used paclitaxel coated balloons as the DEB strategy. New generation thin strut DES or specific everolimus or zotarolimus eluting platforms were most commonly employed, reflecting current PCI practice patterns. DEB therapy was uniformly based on paclitaxel coated balloons, with device details (manufacturer and drug coating) reported consistently but with some variation in stent platforms between studies.

The relative allocation of patients to DES and DEB arms was unbalanced in the retrospective cohorts, with DES used more frequently than DEB (for example, 312 vs. 88 patients in Funayama et al., 409 vs. 219 in Lee et al., 381 vs. 465 in Wańha et al., and 1,478 vs. 138 in Joh et al.). In contrast, the randomized non inferiority trial by Jeger et al. (2018) enrolled similar numbers of patients in each arm (376 DES vs. 382 DEB).

### Clinical outcomes

Because of clinical and methodological heterogeneity and limited number of studies, a formal quantitative meta analysis, subgroup analysis, sensitivity analysis, and publication bias assessment were not performed; instead, outcomes are presented descriptively as in Table 2.

### Target lesion revascularization and target vessel failure

Table 2 showed that Target lesion revascularization (TLR) rates were generally comparable between DEB and DES in studies focusing on dialysis or small vessel disease, but some cohorts suggested differences in target lesion or vessel failure. In the dialysis only cohort by Funayama et al., TLR occurred in 14.1% of DES treated lesions versus 14.7% of DEB treated lesions, with no statistically significant difference (P = 0.864). In the Polish multicenter cohort (Wańha et al., 2024), adjusted analyses favored DES over DEB for TLR, with an adjusted hazard ratio of 0.20 (95% CI 0.06–0.69), indicating a lower risk of repeat revascularization with thin strut new generation DES in that setting.

Target lesion or target vessel failure (TLF/TVF) outcomes showed variable patterns across studies. In Lee et al. (2024), TLF occurred in 13.7% of DES treated patients versus 26.9% of DEB treated patients, corresponding to an adjusted hazard ratio of 0.60 (95% CI 0.27–1.33), suggesting a numerically lower but not statistically significant risk with DES. Conversely, in the cohort by Joh et al. (2024), two year TVF was reported as 27.8% in the DES group compared with 14.3% in the DEB group (HR 2.5, 95% CI 0.29–20), though the wide confidence interval indicates substantial imprecision.

### Major Adverse Cardiovascular Events (MACE)

Two studies explicitly reported MACE as a composite endpoint. In the Japanese dialysis cohort (Funayama et al., 2023), MACE rates were identical between groups (17% in DES vs. 17% in DEB), indicating no apparent difference in composite clinical outcomes in this high risk ESRD population. Similarly, the randomized trial by Jeger et al. (2018) found MACE rates of 10% in both DES and DEB arms, with a hazard ratio of 0.99 (95% CI 0.38–2.57), supporting non inferiority of DEB compared with second generation DES in a mixed coronary population that included patients with renal impairment ( Table 2).

### Thrombosis and other safety outcomes

Data on stent or lesion thrombosis were sparsely reported but showed low event rates overall. In Funayama et al., target lesion thrombosis occurred in 2.2% of DES treated patients and in 0% of DEB treated patients, corresponding to seven events in the DES arm and none in the DEB arm, though the study was not powered to detect differences in rare thrombotic events. Other studies primarily reported composite endpoints such as TLF or TVF and did not consistently separate stent thrombosis or detailed bleeding outcomes in CKD subgroups ( Table 2).

### Summary of risk of bias (Qualitative)

Risk of bias was heterogeneous across the included studies, with four retrospective cohort studies evaluated using ROBINS I and one randomized trial evaluated with RoB 2 ( Table 1). The non randomized cohorts were generally at moderate to serious risk of bias due to confounding, selection of participants, and incomplete adjustment for baseline differences between DEB and DES groups. The randomized trial exhibited low to some concerns of bias in most domains, providing more robust comparative evidence but with limited CKD specific subgroup detail.

## Discussion

This systematic review identified five comparative studies (four retrospective cohort studies and one randomized controlled trial) evaluating drug-eluting balloons (DEB) versus drug-eluting stents (DES) in patients with chronic kidney disease (CKD) or end-stage renal disease (ESRD) undergoing percutaneous coronary intervention (PCI). The five included studies encompassed 4,646 patients across East Asian and European healthcare settings and demonstrated substantial heterogeneity in patient populations, renal function severity, intervention characteristics, and clinical outcomes.

The five included studies demonstrated marked variation in CKD/ESRD representation and severity. Funayama et al. (2023) enrolled exclusively ESRD patients on hemodialysis (100% ESRD, n=400, mean age 69.1 years, 76.3% male), representing the most severely compromised renal population. ^16^ This dialysis-only cohort from Japan provides the most specific evidence for ESRD populations. In contrast, Joh et al. (2024) conducted a large Korean multicenter study (n=1,616, mean age 64.2 years, 78.4% male) but included only 6.7% CKD patients, substantially limiting its generalizability to CKD-specific populations. ^17^ The other three studies represented intermediate CKD severity: Lee et al. (2024) in South Korea explicitly enrolled patients with CKD defined as eGFR <60 mL/min/1.73m ^2^ (28.3% of cohort, n=628), ^18^ while Wańha et al. (2024) in Poland enrolled 18.9% CKD patients (n=846), ^19^ and Jeger et al. (2018) in Switzerland enrolled 14.9% renal failure patients through randomization (n=758, non-inferiority RCT design). ^20^ This heterogeneity in CKD prevalence and definitions (from explicitly defined eGFR thresholds to undefined “renal failure” terminology) substantially limits the ability to draw CKD-specific conclusions from the entire cohort, particularly from the larger studies with lower CKD prevalence. ^17^

All five studies compared paclitaxel-coated balloons (DEB strategy) to contemporary new-generation or second-generation DES. The DES platforms varied: everolimus-eluting stents or zotarolimus-eluting stents, ^17– 19^ paclitaxel-eluting stents, ^16, 20^ and specifically noted thin-strut (<100 μm) new-generation DES in Wańha et al. (2024). ^19^ All DEB arms uniformly employed paclitaxel-coated balloons, reflecting current practice patterns. Study design differences were notable: Jeger et al. (2018) was a randomized controlled trial with non-inferiority design, ^20^ representing the highest level of evidence, while Funayama et al. (2023), Lee et al. (2024), Wańha et al. (2024), and Joh et al. (2024) were retrospective cohort designs. ^16– 19^ Patient allocation to DEB versus DES was unbalanced in the retrospective cohorts, with DES used more frequently than DEB (312 vs. 88 in Funayama; 409 vs. 219 in Lee; 381 vs. 465 in Wańha; 1,478 vs. 138 in Joh), whereas the randomized trial (Jeger) enrolled similar numbers (376 DES vs. 382 DEB). ^16– 19^

TLR and TVF outcomes demonstrated remarkable heterogeneity across the five studies, with conflicting results regarding DEB versus DES superiority. In the dialysis-only Funayama et al. (2023) cohort, TLR rates were nearly identical between groups: DES 14.1% versus DEB 14.7% (P=0.864), indicating no statistically significant difference and suggesting therapeutic equivalence in the most severely compromised renal population. ^16^ However, the Polish multicenter study by Wańha et al. (2024) demonstrated statistical superiority of DES for TLR, with adjusted analyses favoring DES (adjusted hazard ratio 0.2, 95% CI 0.06–0.69), indicating a lower risk of repeat revascularization with thin-strut new-generation DES in that setting. ^19^

TLF/TVF outcomes showed even greater variability. Lee et al. (2024) reported TLF in 13.7% of DES-treated patients versus 26.9% of DEB-treated patients (adjusted hazard ratio 0.60, 95% CI 0.27–1.33), suggesting numerically lower but not statistically significantly lower risk with DES. In marked contrast, the largest cohort by Joh et al. (2024) reported two-year TVF as 27.8% in the DES group compared with 14.3% in the DEB group (HR 2.5, 95% CI 0.29–20), indicating numerically higher TVF with DES, though the extremely wide confidence interval (95% CI spanning 0.29–20) demonstrates substantial imprecision and statistical uncertainty, limiting interpretation. ^17^

Two studies explicitly reported MACE as a composite endpoint, demonstrating clinical equivalence between DEB and DES. In the Japanese dialysis cohort MACE rates were identical between groups: DES 17% versus DEB 17% (P not significant), indicating no apparent difference in composite clinical outcomes in this high-risk ESRD population. ^16^ The randomized trial by Jeger et al. (2018) similarly found MACE rates of 10% in both DES and DEB arms (hazard ratio 0.99, 95% CI 0.38–2.57), supporting non-inferiority of DEB compared with second-generation DES. ^20^ This randomized evidence provides Level 1B quality data suggesting therapeutic equivalence for MACE outcomes in patients with renal impairment.

Data on stent or lesion thrombosis were sparsely reported across the five studies but showed low overall event rates. Funayama et al. (2023) specifically measured target lesion thrombosis and found it occurred in 2.2% of DES-treated patients (7 events) and in 0% of DEB-treated patients, favoring DEB; however, the study was not powered to detect differences in rare thrombotic events. ^16^ The other four studies primarily reported composite endpoints such as TLF or TVF and did not consistently separate or report stent thrombosis, definite or probable stent thrombosis, or detailed bleeding outcomes stratified by CKD subgroups. This sparse thrombosis reporting limits definitive safety conclusions from the current cohort.

In summary, the five included studies demonstrate: (1) clinical equivalence for MACE in high-risk ESRD populations (Funayama) and a randomized trial (Jeger) ^16, 20^; (2) conflicting results for TLR/TVF, with one study favoring DES (Wańha), one favoring DEB numerically (Joh), one favoring DES numerically (Lee), and one showing equivalence (Funayama) ^16– 19^; (3) sparsely reported thrombosis data, with one study showing lower thrombosis with DEB (Funayama) ^16^; and (4) overall heterogeneous outcomes reflecting differences in study design, CKD definitions, intervention platforms, and operator technique. The highest-quality evidence (the Jeger RCT) demonstrated MACE equivalence, while the most CKD-specific evidence (the Funayama dialysis cohort) demonstrated MACE equivalence and TLR equivalence. ^16, 20^

The heterogeneous findings from the current CKD/ESRD cohort contrast with and complement broader evidence from general coronary populations, particularly in the context of small vessel disease. A comprehensive meta-analysis by Sanz Sánchez et al. (2021) of 5 randomized controlled trials (n=1,459) comparing drug eluting balloons (DEB) and drug-eluting stents (DES) in small coronary artery disease found no significant difference in target vessel revascularization (OR 0.97, 95% CI 0.56–1.68, P=0.92), target lesion revascularization (TLR), or all-cause death between the two strategies. ^21^ Importantly, Sanz Sánchez et al. (2021) demonstrated that DEB were associated with a significantly lower risk of vessel thrombosis compared to DES (OR 0.12, 95% CI 0.01–0.94, P=0.04). ^21^ While DES yielded superior acute angiographic results (larger minimal lumen diameter), the clinical equivalence and superior safety profile regarding thrombosis suggest that leaving no permanent metal implant is a viable, and potentially safer, strategy for small vessels—a principle highly relevant to the calcified, diffuse disease often seen in CKD populations.

The current review's findings of MACE equivalence in high-risk ESRD (Funayama, 17% both groups) and the randomized trial (Jeger, 10% both groups) align with Sanz Sánchez et al. (2021)'s demonstration of clinical equivalence in general small-vessel populations. ^16, 20, 21^ However, the conflicting TLR/TVF results in the current CKD studies (ranging from DES superiority in Wańha to DEB numerically better in Joh) diverge from the consistent non-inferiority seen in the general population meta-analyses, suggesting that the uremic milieu may modify the relative efficacy of these devices.

The current review enrolled exclusively CKD/ESRD populations, making the interpretation of their outcomes within the context of how severely renal disease impairs prognosis essential. A comprehensive meta-analysis by Jiang et al. (2023) of 11 studies (n=20,975 patients) examining outcomes after PCI in patients with both diabetes mellitus and CKD found markedly elevated mortality risk: early all-cause mortality was increased 3.45-fold (95% CI 3.07–3.87), late all-cause mortality increased 2.78-fold (95% CI 1.92–4.02), cardiac mortality increased 2.90-fold (95% CI 1.99–4.22), and myocardial infarction risk increased 1.40-fold (95% CI 1.06–1.85) compared to patients without CKD. ^22^ Importantly, Jiang et al. (2023) noted that revascularization rates were not influenced by CKD status, suggesting that CKD primarily affects mortality and ischemic complications rather than the mechanical failure of the revascularization device itself. ^22^

This evidence contextualizes the current review's findings: the absolute MACE rates of 17% (Funayama) and 10% (Jeger) appear reasonable given that Jiang et al. (2023) demonstrated nearly 3-fold mortality increases in CKD populations. ^16, 20, 22^ The finding that device strategy (DEB vs. DES) had minimal impact on MACE in ESRD dialysis patients (Funayama, 17% both groups) is consistent with Jiang et al. (2023)'s conclusion that CKD affects outcomes independently of revascularization technique—suggesting that the systemic uremic condition, rather than device choice, dominates the clinical outcome. ^16, 22^

The mechanism of this poor prognosis is further elucidated by Aoki and Ikari (2017), who describe the unique cardiovascular pathology in patients with end-stage renal disease (ESRD) on hemodialysis. ^23^ They highlight that uremic vasculopathy is characterized by medial hypertrophy, macrophage infiltration, and severe vascular calcification—factors that impair endothelial function and drive restenosis regardless of the device used. Aoki and Ikari (2017) note that while second-generation DES have improved outcomes compared to bare-metal stents, hemodialysis remains a powerful independent predictor of MACE (Hazard Ratios 5.42–5.46 vs non-dialysis), underscoring the inadequacy of local device advancements against the hostile uremic milieu. ^23^ This explains why MACE rates in the current review (10–17%) are higher than general populations (~5–10%) and why device differences are attenuated.

Despite these challenges, the validity of the DEB platform has been reinforced by recent high-quality evidence. The pivotal AGENT IDE randomized clinical trial (Yeh et al., 2024) recently established the superiority of paclitaxel-coated balloons over uncoated balloons for coronary in-stent restenosis (ISR), demonstrating a significant reduction in target lesion failure at 12 months (17.9% vs 28.7%; P=0.006). ^24^ While the AGENT IDE trial focused on ISR, its confirmation of the biological efficacy of paclitaxel transfer in complex lesions supports the rationale for using DEB in the challenging anatomical subsets found in CKD, such as small vessels and diffuse disease, where avoiding additional metal layers is desirable. ^24^

Small vessel disease is particularly prevalent in CKD populations due to chronic ischemia and medial calcification. A recent systematic review and meta-analysis by Murphy et al. (2023) of four randomized controlled trials (BASKET-SMALL 2, PICCOLETO 2, BELLO, RESTORE SVD; n=1,414) specifically compared DEB and DES in small vessel disease. ^15^ Murphy et al. (2023) found: (1) no significant difference in MACE at 1, 2, or 3 years (OR 0.76–0.98); (2) DEB demonstrated significantly lower non-fatal myocardial infarction at 1 year (OR 0.44, 95% CI 0.2–0.94); and (3) BASKET-SMALL 2 demonstrated significantly reduced major bleeding at 2 years with DEB (OR 0.3, 95% CI 0.1–0.91). ^15^

This meta-analytic evidence directly supports several findings in the current review. The MACE equivalence demonstrated in Murphy et al. (2023) mirrors the MACE equivalence found by Funayama in ESRD and Jeger in the RCT. ^15, 16^ The finding of reduced vessel thrombosis by Sanz Sánchez et al. (2021) and reduced MI by Murphy et al. (2023) is consistent with Funayama's report of zero target lesion thrombosis with DEB versus 2.2% with DES. ^15, 16, 21^ Furthermore, the bleeding reduction with DEB (Murphy et al., 2023) is particularly relevant to CKD/ESRD patients who have platelet dysfunction and anticoagulation requirements for hemodialysis, offering a safety advantage even if efficacy is merely equivalent. ^15^

### Limitations of evidence included in the review

Several important limitations affect the strength of conclusions from this systematic review. First, methodological heterogeneity is substantial: four studies employed retrospective cohort designs with inherent selection bias and confounding, while only one randomized trial with explicit non-inferiority endpoints was included. ^20^ Only the Jeger et al. (2018) study employed randomization; the remaining studies lacked propensity-score matching or multivariable adjustment for baseline differences, limiting causal inference. ^18^

Second, CKD/ESRD definition and prevalence varied markedly across studies. Funayama et al. (2023) enrolled exclusively dialysis-dependent ESRD patients (100%), while Joh et al. (2024) included only 6.7% CKD prevalence, substantially limiting the ability to draw CKD-specific conclusions from the largest cohort. ^16, 17^ CKD definitions ranged from explicitly defined eGFR <60 mL/min/1.73m ^2^ (Lee et al., 2024, using the four-component MDRD equation) to undefined “renal failure” terminology, ^19, 20^ precluding severity-based subgroup analysis.

Third, outcome definitions and follow-up durations differed substantively among studies. Target lesion revascularization, target vessel failure, and MACE definitions varied, with heterogeneous follow-up durations limiting meta-analytic synthesis. The absence of standardized outcome definitions prevented quantitative pooling of results.

Fourth, four cohorts were explicitly at “moderate” risk of bias using ROBINS-I assessment, primarily due to confounding and selection bias from non-randomized designs. The unbalanced allocation of patients to DEB versus DES in retrospective cohorts (particularly extreme in Joh et al. with 1,478 DES vs. 138 DEB) introduces allocation bias and confounding by indication, as operators may have selected devices based on lesion characteristics or patient factors not uniformly reported.

Fifth, stent platform heterogeneity confounds interpretation. While all DEB arms used paclitaxel-coated balloons, DES platforms included both everolimus-eluting and zotarolimus-eluting second-generation devices (Lee et al., 2024; Wańha et al., 2024; Joh et al., 2024) and paclitaxel-eluting stents (Jeger et al., 2018; Funayama et al., 2023), introducing device-specific efficacy variation. ^16– 20^ The single RCT (Jeger et al., 2018) compared paclitaxel-coated balloons to everolimus-eluting or paclitaxel-eluting second-generation DES, while retrospective cohorts may have included diverse or older-generation stent platforms not specified in baseline characteristics. ^20^

Sixth, insufficient CKD-subgroup analysis within studies limits interpretation. While all studies enrolled CKD/ESRD patients, none performed prespecified subgroup analysis stratified by CKD stage (eGFR thresholds), dialysis dependence, or baseline renal function, precluding assessment of whether DEB or DES advantage/disadvantage varies with renal dysfunction severity.

Seventh, procedural variables potentially influencing outcomes were incompletely reported. Critical DEB deployment parameters (transit time, balloon-to-artery ratio, inflation pressure) were not consistently documented in the studies, limiting assessment of whether technical factors—known from Villar-Matamoros et al. (2022) to substantially affect paclitaxel delivery—explained outcome heterogeneity. ^25^ Similarly, predilation adequacy, intravascular ultrasound guidance, and rotational atherectomy use for calcific lesions were not uniformly reported, yet substantially influence restenosis risk in calcified CKD vessels based on Murphy et al. (2023)'s findings of 5.2–34.5% dissection rates depending on technique. ^15^

### Limitations of review processes

The review methodology employed PRISMA 2020 guidelines but faced inherent limitations. Formal quantitative meta-analysis was explicitly not performed due to clinical and methodological heterogeneity, instead employing qualitative synthesis with descriptive outcome tabulation. This conservative approach prevents meta-analytic bias but sacrifices statistical power to detect true treatment effects. Publication bias assessment was not undertaken, limiting confidence that published results represent all conducted analyses. The review did not perform GRADE certainty-of-evidence assessment, which would provide transparency regarding confidence in summary estimates. Only five studies met inclusion criteria despite screening 4,093 initial records, resulting in a limited evidence base. Furthermore, no authors were contacted for unreported subgroup analyses or missing data, potentially introducing information loss. The review's geographic limitation to East Asian and European cohorts may limit generalizability to other healthcare settings with different interventional practices, stent availability, or antiplatelet therapy protocols.

### Implications for practice, policy, and future research

**Clinical practice implications**

Current findings confirm MACE equivalence between DEB and DES in high-risk ESRD (Funayama et al.: 17% both) and renal-inclusive RCT (Jeger et al.: 10% both), corroborating Aoki et al. (2017)'s assertion of uremic pathophysiology's dominance over device choice. ^16, 20, 23^ In moderate-severe CKD/ESRD undergoing PCI, prioritize lesion morphology, vessel size, and bleeding risk over renal status alone, as both modalities yield comparable MACE and mortality. DEB noninferiority to DES in small vessels (<2.75–3 mm), with DEB advantages in myocardial infarction prevention, bleeding mitigation, and abbreviated dual antiplatelet therapy (1–3 vs. 6–12 months)—pertinent for uremic platelet dysfunction and hemodialysis anticoagulation; ESRD thrombosis disparity (Funayama: 0% DEB vs. 2.2% DES) reinforces this. ^16^

**Policy implications**

The current review and literature advocate CKD/ESRD-specific PCI registries stratifying DEB versus DES outcomes with standardized metrics and staging to yield pragmatic evidence beyond scarce RCTs. Mandate operator training/certification for DEB in complex calcified CKD anatomy, given technique. Implement reimbursement parity for DEB matching DES, reflecting equivalent/superior CKD efficacy—especially bleeding/small-vessel benefits—despite current disparities.

**Future research directions**

Prospective RCTs enrolling CKD/ESRD patients with eGFR (<15, 15–29, 30–59 mL/min/1.73 m ^2^), dialysis, and calcification stratification (SYNTAX score) are essential to compare contemporary DEB/DES, standardizing MACE, dual antiplatelet therapy, and hemodialysis anticoagulation while capturing bleeding/thrombosis/revascularization endpoints. Mechanistic inquiries should probe uremic endothelial recovery kinetics post-DEB/DES, neointimal histology in CKD restenosis models, and paclitaxel responses in smooth muscle cells to discern if DEB scaffold avoidance enhances healing amid 2.78–3.45-fold CKD mortality escalation.

Ex vivo/in vivo pharmacokinetic assessments of paclitaxel delivery/retention in calcified vs. non-calcified CKD vessels via intravascular ultrasound/optical coherence tomography will guide predilation/balloon sizing for DEB comparability to DES in heavy calcification. Health economic evaluations must quantify DEB vs. DES cost-effectiveness incorporating antiplatelet/bleeding/hospitalization/quality-of-life burdens, alongside registries with uniform MACE/KDIGO criteria and CKD subgroups for pragmatic insights. Technical trials optimizing DEB predilation (intravascular ultrasound), ratios/pressures/inflation in stratified CKD/calcification will mitigate heterogeneity.

## Conclusion

This systematic review indicates that in patients with chronic kidney disease (CKD) and end-stage renal disease (ESRD) undergoing percutaneous coronary intervention, drug-coated balloons and second-generation drug-eluting stents provide comparable outcomes for major adverse cardiovascular events at intermediate-term follow-up. These findings suggest that, in advanced renal disease, systemic factors related to the uremic milieu may attenuate device-specific differences in clinical outcomes.

In contrast, outcomes related to target lesion or vessel failure remain heterogeneous and context-dependent, precluding definitive conclusions regarding the superiority of either strategy across all CKD populations. This variability likely reflects differences in study design, renal disease severity, lesion characteristics, and procedural practice.

Overall, the available evidence supports an individualized revascularization approach in CKD/ESRD patients, in which device selection should be guided by patient risk profile, lesion morphology, bleeding risk, and operator expertise rather than renal status alone. Further CKD-specific prospective randomized studies with standardized outcome definitions and procedural protocols are required to refine optimal revascularization strategies in this high-risk population.

## Data Availability

All data underlying the results are available in Kaggle under CC0 license ( https://doi.org/10.34740/KAGGLE/DSV/14546872). ^26^ PRISMA checklist and flowchart for ‘Comparative Outcomes of Drug-Eluting Stents Versus Drug-Eluting Balloons in Patients with Chronic Kidney Disease: A Systematic Review of Current Evidence’ available on Kaggle under CC0 license. ^27, 28^ The DOI for PRISMA checklist is https://doi.org/10.34740/KAGGLE/DSV/14546642. While DOI for PRISMA flowchart is https://doi.org/10.34740/KAGGLE/DSV/14555295.
