# Supplementary material for: Comparative Outcomes of Drug-Eluting Stents Versus Drug-Eluting Balloons in Patients with Chronic Kidney Disease: A Systematic Review of Current Evidence
Source: F1000Res. 2026 Feb 18;15:287. [Version 1] doi: 10.12688/f1000research.177235.1 (PMC12966798; doi:10.12688/f1000research.177235.1)
Supplement: Peer review history

# Reviewer report: Reviewer response for version 1

**DOI:** 10.5256/f1000research.195427.r460225

**Published:** 2026-03-06

**Authors:** Amja Manullang

**Affiliations:** 1 Middlesex University, London, UK

This study presents a systematic review comparing the efficacy and safety of Drug-Eluting Stents and Drug-Eluting Balloons in patients with Chronic Kidney Disease (CKD). Overall, the review follows the PRISMA reporting guidelines. However, the manuscript requires minor improvements in clarity, as follows:

1. In the introduction section, the authors state that long-term data remain scarce and the durability of DEB outcomes beyond the medium-term follow-up period is still uncertain. Therefore, it would be helpful to briefly summarize the follow-up periods of the five included studies in the Results or Discussion section.
2. Please clarify whether there was any restriction on the publication years in the inclusion criteria.
3. I suggest removing database categories with zero results (n = 0) from the PRISMA flowchart, such as Registers (n = 0), records marked as ineligible by automation tools (n = 0), records removed for other reasons (n = 0), and reports not retrieved (n = 0), reports of new included studies (n=0)
4. Please provide explanations of abbreviations below Table 2, such as CI (confidence interval), HR (hazard ratio), etc.

Are the rationale for, and objectives of, the Systematic Review clearly stated?

Yes

Is the statistical analysis and its interpretation appropriate?

Yes

If this is a Living Systematic Review, is the ‘living’ method appropriate and is the search schedule clearly defined and justified? (‘Living Systematic Review’ or a variation of this term should be included in the title.)

Not applicable

Are sufficient details of the methods and analysis provided to allow replication by others?

Yes

Are the conclusions drawn adequately supported by the results presented in the review?

Yes

Reviewer Expertise:

Systematic Review and Meta-Analysis

I confirm that I have read this submission and believe that I have an appropriate level of expertise to confirm that it is of an acceptable scientific standard.

# Reviewer report: Reviewer response for version 1

**DOI:** 10.5256/f1000research.195427.r460223

**Published:** 2026-02-25

**Authors:** Indra Prasetya

**Affiliations:** 1 Universitas Brawijaya, Malang, Indonesia

The authors present a systematic review comparing clinical outcomes of drug-eluting balloons (DEB) and drug-eluting stents (DES) in patients with chronic kidney disease (CKD) or end-stage renal disease (ESRD) undergoing percutaneous coronary intervention. The review includes five comparative studies—four retrospective cohorts and one randomized controlled trial—encompassing a total of 4,646 patients.

 Given the substantial heterogeneity across studies in terms of CKD definitions, disease severity, study design, and device platforms, the authors appropriately chose a narrative synthesis rather than a quantitative meta-analysis. Overall, the review suggests comparable rates of major adverse cardiovascular events (MACE) between DEB and DES, while outcomes related to target lesion and target vessel failure remain inconsistent across studies. Based on these findings, the authors recommend an individualized revascularization approach that considers lesion characteristics and bleeding risk rather than renal status alone.

**Detailed Feedback**

**1. Rationale and Objectives**

 The rationale for this review is clearly and convincingly presented. The authors appropriately emphasize that patients with CKD face a higher risk of adverse cardiovascular outcomes and bleeding complications following PCI, and that long-term outcomes with DES remain suboptimal in this population. The introduction clearly explains why DEB, by avoiding permanent metal implantation, represents a potentially attractive alternative. The study objectives follow logically from this background and are clearly stated.

**2. Methods and Analysis**

 The methodology is well described and allows for reproducibility. The review was prospectively registered in PROSPERO (CRD420251039001) and conducted in accordance with PRISMA 2020 guidelines. The literature search strategy is comprehensive, covering multiple major databases up to September 2025, and the processes for study selection, data extraction, and risk-of-bias assessment are clearly outlined. The use of validated tools (RoB 2 and ROBINS-I) further strengthens the methodological rigor.

**3. Statistical Analysis and Interpretation**

 The decision not to perform a meta-analysis is justified and appropriate. The included studies differ substantially in CKD definitions (ranging from non-dialysis CKD to 100% dialysis-dependent ESRD), study design, and interventional platforms. Pooling these heterogeneous data would risk producing misleading summary estimates. The narrative synthesis is structured and balanced, and the authors avoid overstating the strength of the available evidence.

**4. Conclusions**

 The conclusions are supported by the data presented. The finding of MACE equivalence is mainly driven by one randomized trial and one ESRD dialysis cohort, and this is appropriately acknowledged. The authors also correctly highlight the inconsistent findings for TLR and TVF and place these results within the broader context of uremic cardiovascular pathology, where systemic factors may outweigh device-specific effects. The recommendation for individualized treatment is reasonable and clinically sensible.

**Constructive Suggestions and Minor Revisions**

 While the manuscript is scientifically sound and suitable for approval, a few minor revisions would further improve clarity:

- **Abstract clarity:** One of the largest included cohorts (Joh et al., 2024; n=1,616) had a CKD prevalence of only 6.7%. Although this limitation is discussed in the main text, briefly noting in the Abstract that CKD prevalence varied widely across studies would help avoid the impression that all 4,646 patients had CKD.
- **Grammatical correction:** In the Abstract (Methods section), “cohort study” should be corrected to “cohort studies,” as four retrospective cohorts were included.

Are the rationale for, and objectives of, the Systematic Review clearly stated?

Yes

Is the statistical analysis and its interpretation appropriate?

Yes

If this is a Living Systematic Review, is the ‘living’ method appropriate and is the search schedule clearly defined and justified? (‘Living Systematic Review’ or a variation of this term should be included in the title.)

Not applicable

Are sufficient details of the methods and analysis provided to allow replication by others?

Yes

Are the conclusions drawn adequately supported by the results presented in the review?

Yes

Reviewer Expertise:

Interventional Cardiology, Cardiac Intensive Care, and Percutaneous Coronary Intervention (PCI).

I confirm that I have read this submission and believe that I have an appropriate level of expertise to confirm that it is of an acceptable scientific standard.
